# A new model for non-typeable *Haemophilus influenzae* middle ear infection in the *Junbo* mutant mouse

**DOI:** 10.1242/dmm.021659

**Published:** 2016-01-01

**Authors:** Derek Hood, Richard Moxon, Tom Purnell, Caroline Richter, Debbie Williams, Ali Azar, Michael Crompton, Sara Wells, Martin Fray, Steve D. M. Brown, Michael T. Cheeseman

**Affiliations:** 1 MRC Mammalian Genetics Unit, MRC Harwell, Didcot, Oxford, OX11 0RD, UK; 2 Department of Paediatrics, University of Oxford Medical Sciences Division, John Radcliffe Hospital, Oxford, OX3 9DU, UK; 3 Developmental Biology Division, The Roslin Institute and Royal (Dick) School of Veterinary Studies, Easter Bush Campus, University of Edinburgh, EH25 9RG, UK; 4 Mary Lyon Centre, MRC Harwell, Harwell, Didcot, Oxford, OX11 0RD, UK

**Keywords:** Azithromycin, *Junbo* mouse, Non-typeable *Haemophilus influenzae*, Immunization, Otitis media

## Abstract

Acute otitis media, inflammation of the middle ear, is the most common bacterial infection in children and, as a consequence, is the most common reason for antimicrobial prescription to this age group. There is currently no effective vaccine for the principal pathogen involved, non-typeable *Haemophilus influenzae* (NTHi). The most frequently used and widely accepted experimental animal model of middle ear infection is in chinchillas, but mice and gerbils have also been used. We have established a robust model of middle ear infection by NTHi in the *Junbo* mouse, a mutant mouse line that spontaneously develops chronic middle ear inflammation in specific pathogen-free conditions. The heterozygote *Junbo* mouse (*Jbo/+*) bears a mutation in a gene (*Evi1*, also known as *Mecom*) that plays a role in host innate immune regulation; pre-existing middle ear inflammation promotes NTHi middle ear infection. A single intranasal inoculation with NTHi produces high rates (up to 90%) of middle ear infection and bacterial titres (10^4^-10^5^ colony-forming units/µl) in bulla fluids. Bacteria are cleared from the majority of middle ears between day 21 and 35 post-inoculation but remain in approximately 20% of middle ears at least up to day 56 post-infection. The expression of Toll-like receptor-dependent response cytokine genes is elevated in the middle ear of the *Jbo*/+ mouse following NTHi infection. The translational potential of the *Junbo* model for studying antimicrobial intervention regimens was shown using a 3 day course of azithromycin to clear NTHi infection, and its potential use in vaccine development studies was shown by demonstrating protection in mice immunized with killed homologous, but not heterologous, NTHi bacteria.

## Abstract (precis)

**Summary:** Acute otitis media is an important disease in children. We describe a new infection model for translational research that uses the *Junbo* mouse mutant intranasally inoculated with non-typeable *Haemophilus influenzae*.

## INTRODUCTION

Acute otitis media (AOM) is primarily caused by the bacterial commensal pathogens non-typeable *Haemophilus influenzae* (NTHi), *Streptococcus pneumoniae* (pneumococcus) and *Moraxella catarrhalis*. AOM is the most common bacterial infection in children and, as a consequence, is the commonest reason for antibiotic prescription during childhood, accounting for 24.5 million physician office visits per year in the USA (Bakaletz, 2009). The global health burden of AOM is significant; of an estimated 709 million cases per year, 31 million children go on to develop chronic suppurative otitis media (otitis media with otorrhoea; i.e. perforation of the tympanic membrane and drainage of pus), and an estimated 21,000 deaths occur each year from complications such as meningitis (Monasta et al., 2012).

The chinchilla otitis media (OM) model was first developed to study pneumococcus infection (Giebink et al., 1976). It has subsequently become the most frequently used model for NTHi infection studies and vaccine development, and is considered to be a robust, reproducible model for polymicrobial infections (Bakaletz, 2009). Following direct inoculation of the chinchilla bulla, NTHi can form a biofilm that promotes bacterial survival against the host response and treatment (Jurcisek and Bakaletz, 2007; Brockson et al., 2014). In addition to the chinchilla, there is increasing use of inbred strains of mice and mouse mutants in OM research (Ryan et al., 2006; Sabirov and Metzger, 2008; Hernandez et al., 2015). In chinchillas and mice, direct injection of bacteria into the middle ear (ME) bulla is efficient and allows the dose to be controlled, but intranasal (IN) inoculation that mimics natural ascending Eustachian tube (ET) infection produces only sporadic ME infection in mice (Ryan et al., 2006) and no ME infection in chinchillas (Bakaletz, 2009). However, IN inoculation of the chinchilla produces sustained infection of the ME when it is used in conjunction with barotrauma (lowering pressure in the ME; Giebink et al., 1979) or following virus infection (Giebink, 1981). In mice, IN inoculation results in colonization of the nasopharynx (NP), and subsequent ME infection is enhanced by repeated IN inoculation (Sabirov and Metzger, 2006), when IN inoculation is used in conjunction with virus infection (McCullers et al., 2007; Short et al., 2013) or barotrauma (by increasing the environmental air pressure; Stol et al., 2009).

Mice with mutations in transforming growth factor-β signalling pathway genes that modulate pro-inflammatory responses or with mutations that lead to ET malformation are predisposed to develop OM spontaneously without the need for bacterial challenge, and a number of these genes are also implicated in modulating susceptibility to human OM (review by Rye et al., 2010).

In this study, we focus on NTHi-induced ME infection in the *Junbo* mouse, a mutant mouse line that spontaneously develops chronic ME inflammation in specific pathogen-free (SPF) conditions. The heterozygote *Junbo* mouse (*Jbo*/*+*) bears an Asn763Ile mutation in the gene encoding the transcription factor Evi1, also known as Mecom (Parkinson et al., 2006). One mechanism that might underlie the predisposition to OM in *Jbo*/*+* mice is that Evi1 is a negative regulator of nuclear factor-κB, and the loss-of-function *Evi1* *Junbo* mutation exacerbates NTHi-induced inflammation in the lung (Xu et al., 2012).

We hypothesized that the ME inflammation in *Jbo*/*+* mice could provide a niche in which, after IN challenge, bacteria would establish infection following contiguous spread along the ET. As a proof of this concept and its utility as a validated animal model of OM, we used the human commensal pathogen NTHi to establish ME infection. We have characterized the dynamics and host responses to NP colonization and OM, using multiple, genetically distinct NTHi strains. Our data demonstrate the utility of the *Jbo*/*+* model for testing immunization and antibiotic strategies aimed at the prevention or treatment of NTHi infection of the ME.

## RESULTS

### NTHi inoculation intranasally infects the ME of *Jbo*/*+* mice for at least 56 days

**Fig. 1.**
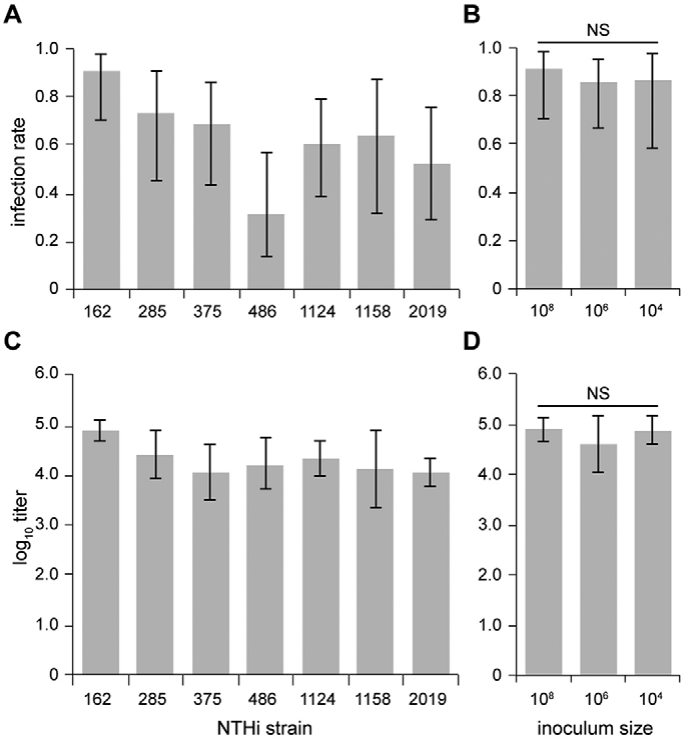
**IN inoculation with different NTHi strains produces variation in ME infection rate and bacterial titre, but for NTHi 162 these parameters do not vary over a 10^4^-10^8^ CFU inoculum dose.** (A,C) *Jbo*/*+* mice were sampled 7 days after IN inoculation with different NTHi strains; ME infection rates (A) and titres (C) vary with NTHi strain. (B,D) For NTHi 162, ME infection rate (B) and titres (D) were not significantly different when inoculum doses were used over the range of 10^4^-10^8^ CFU. *n*=11-12 mice per cohort; data are represented as mean±95% CI. Fisher’s exact test in B; one-way ANOVA in D; NS, not significant.

We have successfully established a robust model of ME infection using a single IN inoculation with the commensal pathogen NTHi in the *Junbo* mouse. Seven well-studied and unrelated NTHi isolates from human OM and one from lower respiratory tract disease (strain 2019) were inoculated IN [10^8^ colony-forming units (CFU)] into 8-week-old SPF *Jbo*/+ mice, then sampled after culling on day 7 post-infection. For seven of the NTHi isolates, high bacterial titres of 10^4^-10^5^ CFU/µl were obtained from 30-90% of infected ME bullae (Fig. 1A,C). Infection with NTHi 176 was unsuccessful. Strains NTHi 162 and 375 were chosen as the principal test strains for subsequent experiments.

The ME infection rates (Fig. 1B) and titres (Fig. 1D) were not significantly different when inocula ranging from 10^4^ to 10^8^ CFU of NTHi 162 were used. Following an IN challenge of 10^3^ CFU, a significant increase in ME NTHi load 7 days post-infection was detected [10^4.7^ CFU/µl, 95% confidence interval (CI) 10^4.2^-10^5.3^, *n*=7, *P*=0.000185, one-sample *t*-test]; however, the ME infection rate was reduced with this low inoculum size (24 versus 75% for 10^3^ and 10^6^ CFU 162sr inoculation groups (*n*=15), respectively, *P*=0.000178).

**Fig. 2.**
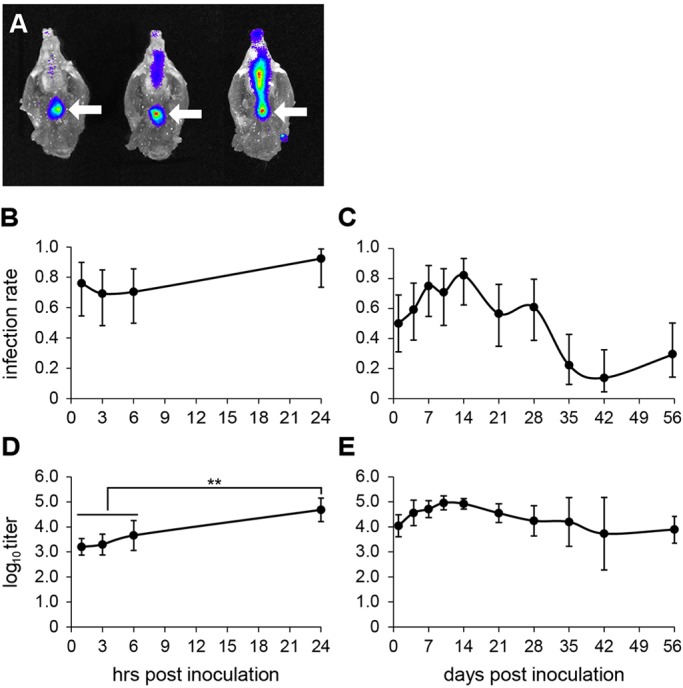
**Time course of NTHi ME infection.** (A) A bioluminescent signal from 10^8^ CFU NTHi 162lux is evident level with opening of the NP (arrow) minutes after IN inoculation of three *Jbo*/*+* mice. (B,D) A 24 h time course for ME infection rate (B) and log_10_ titres (D) in 12-week-old *Jbo*/*+* mice inoculated with 10^6^ CFU of NTHi 162sr (detection limit of 10 CFU/µl). (C,E) Extended time course from 1 to 56 days post-inoculation (detection limit of 100 CFU/µl) for ME infection rate (C) and log_10_ titres (E). *n*=13-15 mice per time point; data are represented as mean±95% CI. (D) One-way ANOVA *P*<0.001; using Tukey's multiple comparison tests, the NTHi titres at 1, 3 and 6 h are not significantly different from one another, but each is significantly different from the NTHi titre at 24 h (**P<0.01).

The potential for rapid transfer of bacteria from the NP to the ME was demonstrated following IN inoculation with 10^8^ CFU of strain NTHi 162lux. Within minutes, a bioluminescent signal was distributed along the full length of the NP (Fig. 2A), and NTHi were adjacent to the ET opening even though the inoculation volume (10 µl) was small relative to that of the nasal cavity (30 µl; Maronpot et al., 1999). Subsequent spread and dilution of the inoculum resulted in insufficient signal to permit direct monitoring of bacterial movement from the NP. We therefore administered IN 10^8^ fluorescein isothiocyanate (FITC)-labelled 1 µm microspheres as a marker for ET ascension. Five minutes after administration, the numbers of microspheres in histological sections of the ET were not significantly different between 9-week-old *Jbo*/+ mice (median 28/mm, 95% CI 12-77) and wild-type littermate mice (median 29/mm, 95% CI 8-39; *P*=0.6569).

We examined ascending ET infection in 8-week-old *Jbo*/+ mice by sampling bulla fluids and detected NTHi at 1, 3, 6 and 24 h after IN inoculation with 10^6^ CFU of NTHi strain 162sr. With a 10 CFU/µl detection limit for NTHi, ME bulla fluid infection rates were high (≥70%) from 1 h onwards (Fig. 2B). ME NTHi titres were 10^3.2^-10^3.7^ CFU/µl at 1-6 h and increased significantly to 10^4.7^ CFU/µl (95% CI 10^4.2^-10^5.2^) by 24 h post-inoculation (Fig. 2D). Over a 56 day time course, ME infection rates were consistently high (≥60%) 4-14 days post-inoculation, then declined to ∼20% and were maintained at this rate over the period from day 35 to 56 (Fig. 2C). ME titres peaked at day 10 and remained at ∼10^4^-10^5^ CFU/µl until day 56 (Fig. 2E), the last period sampled.

There was a strong positive association (*P*=2.62×10^−16^) between NTHi infection in the ME and NP (Table S1) and ME-NP co-infection declines with time (Fig. S1). The recovery of NTHi by NP washing is only semi-quantitative, and the counts were generally low, 10^1.1^ CFU (95% CI 10^0.9^-10^1.1^, *n*=86 in 50 µl sample of 200 µl wash volume).

**Fig. 3.**
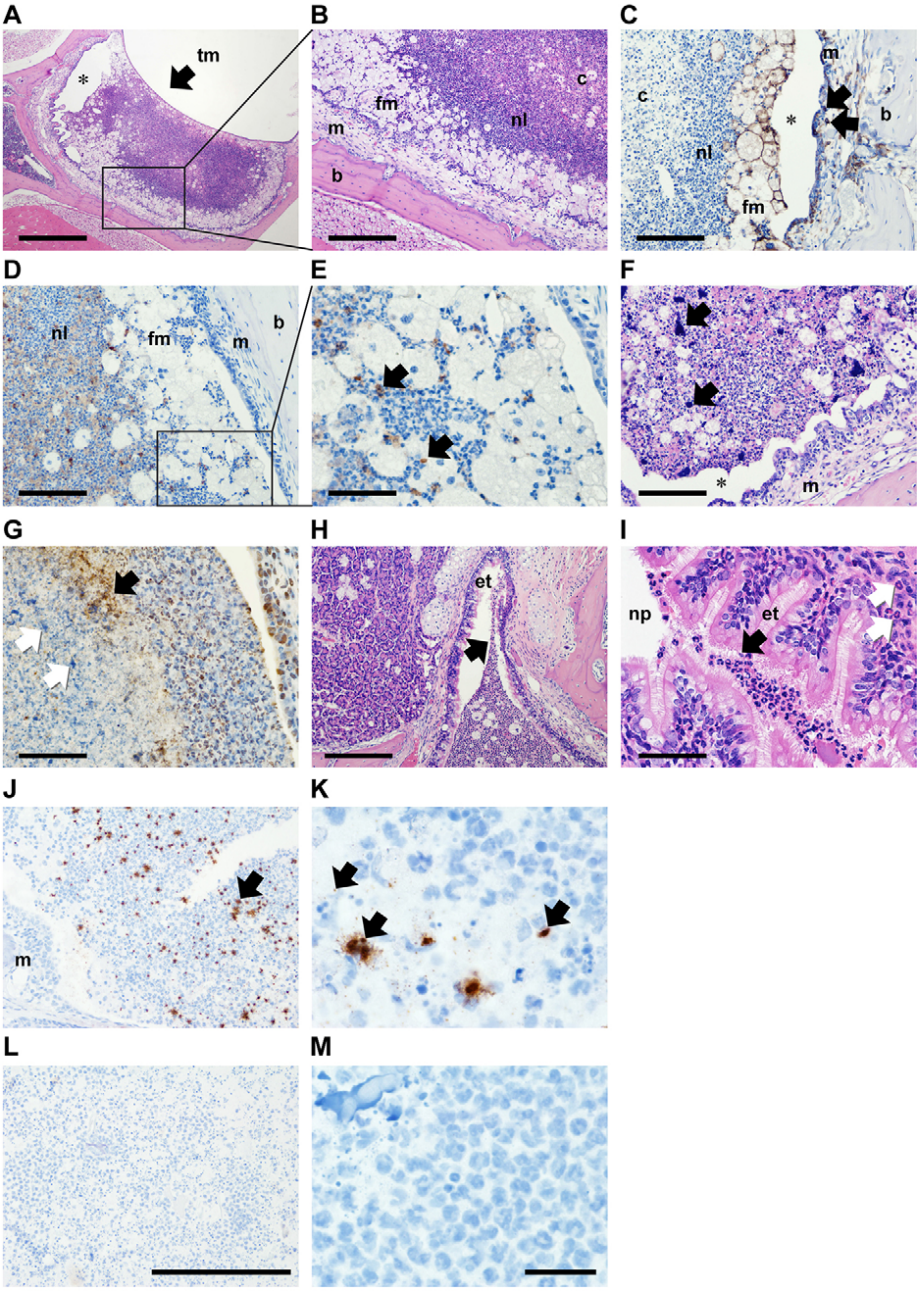
**Histopathology of the *Jbo*/*+* mouse ME 7 days after inoculation with NTHi.** Composite histological features from three 12-week-old *Jbo*/*+* mice 7 days post IN-inoculation with 10^6^ CFU NTHi 162. (A) Haematoxylin- and Eosin-stained section of ME bulla, showing the tympanic membrane (tm; arrow). (B) Higher-magnification view (boxed area in A). Bulla fluid has a caseous core of necrotic neutrophil leukocytes (c) surrounded by viable neutrophil leukocytes (nl) and foamy macrophages (fm); inflamed ME mucosa (m) and bulla bone (b). (C) F4/80 antibody-positive foamy macrophages in bulla fluid (fm) and macrophages in the inflamed mucosa (arrows). (D,E) Cleaved caspase 3-positive apoptotic cells (arrows in E, which is a higher magnification view of the boxed area in D). (F) Two of six bulla fluids had large extracellular accumulations of Haematoxylin-positive chromatin (arrows). (G) The larger chromatin foci were histone 3 antibody negative (white arrows), but finely granular extracellular histone 3-positive material (black arrow) was scattered in the caseous core. Note normal histone staining of neutrophil and epithelial nuclei. (H,I) Neutrophils in Eustachian tube (et) lumen adjacent to the bulla opening (H) and at the nasopharynx (np) junction (I; black arrows), neutrophil leukocyte infiltration in ET submucosa (white arrows). (J,K) *Jbo*/*+* mouse inoculated with NTHi 162sr, probed with B-HInfluenzae-NTHi375-16SrRNA oligonucleotide (Advanced Cell Diagnostic) and visualized using HRP and DAB. Bulla fluid exudates have chromogen deposits (arrows) that appeared as scattered punctate signals (∼1 μm) or irregular aggregates (10-20 μm greatest dimension); the mucosal margin is marked (m). (L,M) NTHi hybridization signals were absent in non-inoculated *Jbo*/*+* control mice. A positive control probe (Ppib) for mouse RNA integrity showed punctate signals in mucosal epithelium and bulla fluid cells, but no signal was obtained with a negative control probe (bacterial DapB gene); data not shown. Scale bars: 500 µm in A, 200 µm in B,H, 100 µm in C,D,F and 50 µm in E,G,I. In A,C,F, asterisk indicates an artefactual cleft caused by histological processing. (J,L) Magnification ×200; scale bar: 200 µm. (K,M) ×1000 oil immersion; scale bar: 20 µm.

The histology of the NTHi-infected middle ear was examined in 12-week-old *Jbo*/*+* mice at day 7 post IN inoculation. To maintain the anatomical integrity of the bulla contents, the tympanic membrane (TM) was not opened. A necrotic caseous core of neutrophils was surrounded by viable and apoptotic neutrophils (cleaved caspase 3 positive) and an outer, variably thick, band of foamy macrophages (F4/80 positive; Fig. 3A-E). There were variable amounts of amorphous extracellular chromatin within the caseous areas (Fig. 3F). The larger accumulations were histone 3 negative, but smaller granular aggregates were histone 3 positive (Fig. 3G). Neutrophil leukocytes were present in the ET lumen and ET mucosa at its junction with the NP and in the ET lumen where it opens into the ME (Fig. 3H,I). The NP mucosa adjacent to the ET opening was not inflamed. Taken together, these data are consistent with an interpretation that the relatively large NTHi populations in the ME act as a reservoir for reinfection through contiguous descending spread from the ME to the NP along with efflux of exudate. The overall histology of chronic OM in 12-week-old *Jbo*/+ mice was not significantly different between NTHi-challenged and non-challenged mice. Sixty (median; 95% CI, 50-75) and 67% (33-81; *P*=0.5339) of the bulla was occupied by neutrophils and foamy macrophages, and the thickness of middle ear mucosa was 100 (84-104) and 111 µm (79-150; *P*=0.086) in NTHi-challenged and non-challenged *Jbo*/+ mice, respectively.

Gram staining failed to reveal Gram-negative coccobacilli in foamy macrophages, and the presence of abundant karyorrhectic nuclear debris in the necrotic core confounded unequivocal demonstration of Gram-negative bacteria in the centre of the mass. To localize NTHi bacteria in the bulla, we used *in situ* hybridization targeting the 16S rRNA of NTHi, and this gave strong signals from the bulla exudate but not elsewhere in head tissues from *Jbo*/*+* mice challenged with NTHi 162. NTHi hybridization signals comprised punctate or larger aggregates and were scattered throughout bulla exudate but were less frequent in the caseous core (Fig. 3J,K). Evidence consistent with the presence of significant or mature biofilm was not found. By contrast, NTHi hybridization signals were absent in non-challenged *Jbo*/*+* control mice (Fig. 3L,M).

### Bulla fluids are critical for NTHi infection in different mouse backgrounds

In NTHi-inoculated 8-week-old wild-type mice, 97.2% (71 of 73) had healthy ears (a clear TM with no detectable intrabulla fluid) and PBS washes were NTHi culture negative. Only two (of 73) NTHi-inoculated wild-type mice had OM; both had unilateral disease and the bulla fluids were NTHi culture positive, indicating that NTHi infection of the wild-type mouse ME was indeed a rare event. A proportion of NTHi-inoculated *Jbo*/+ mice had unilateral or no OM (see below). Only 1.4% (three of 206) of PBS washes from these healthy *Jbo*/+ ears gave NTHi-positive cultures.

NTHi infection in the *Jbo*/*+* mouse did not alter the frequency of OM. The incidence of ME bulla fluids, judged by TM opacity, in *n*=81 NTHi-challenged *Jbo*/*+* mice aged 8 weeks and inoculated with 10^8^ CFU of NTHi (pooled data from experiments with seven NTHi strains) and sampled 7 days later was not significantly different from previously published data on non-challenged 8-week-old *Jbo*/*+* mice (*n*=54; Cheeseman et al., 2011); bilateral OM 85.1 versus 77.8%, unilateral OM 13.6 versus 13.0%, no macroscopic ME disease 1.2 versus 9.3%, each respectively (*P*=0.10 2×3 Fisher’s exact test).

Eight-week-old *Jbo*/*+* mice congenic on a BALB/c background also had a spontaneous OM phenotype, and NTHi ME infection rates and titres were similar to those seen in *Jbo*/*+* mice congenic on the standard C3H/HeH background used in this study. For other mutant mouse lines with a spontaneous OM phenotype when infected by NTHi, 8- to 14-week-old *Tgif* (*Tgif−/−*; Tateossian et al., 2013) has lower ME titres and 7- to 8-week-old *Jeff* (*Jf/+*; Hardisty-Hughes et al., 2006) have the lowest ME titres and infection rates compared with *Jbo*/*+* (Fig. S2). Together, these results indicate that pre-existing inflammation and ME bulla fluid are necessary requirements for NTHi colonization and infection. IN inoculation with NTHi does not induce OM efficiently in the healthy fluid-free ME of either wild-type or *Jbo*/*+* mice nor does it increase the incidence of bulla fluids in *Jbo*/*+* mice. Furthermore, mice with different OM-related mutations behave differently with respect to NTHi infection and do not simply facilitate a non-specific access of bacteria to infect the ME.

### The role of indigenous mouse ME flora

**Fig. 4.**
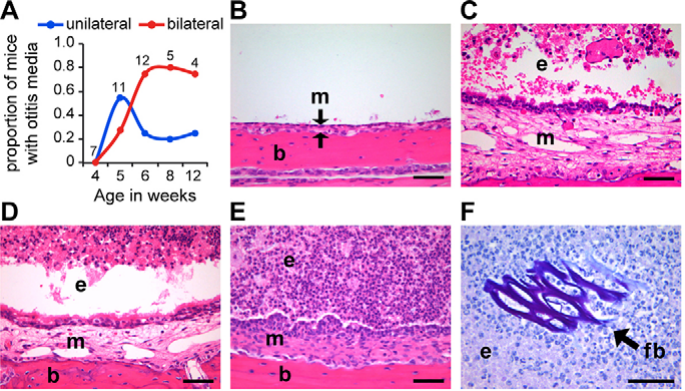
***Jbo*/*+* mice develop OM in GF conditions.** (A) Time course of the proportion of *Jbo*/*+* GF mice with unilateral or bilateral OM (the number of mice is indicated alongside each time point). (B-F) Histological analysis of *Jbo*/*+* mouse ME. (B) Haematoxylin- and Eosin-stained sections of non-inflamed ME of a 4-week-old GF *Jbo*/*+* mouse with an air-filled bulla space, thin mucosa (m) indicated between arrows, supported by underlying bulla bone (b). (C) Inflamed ME of a 4-week-old SPF *Jbo*/*+* mouse with exudate (e) in the bulla space and thickened mucosa (m). (D,E) Inflamed ME of GF *Jbo*/*+* with bulla exudate (e) and thickened mucosa (m) at 5 (D) and 8 weeks of age (E). (F) ME exudate in a 12-week-old GF *Jbo*/*+* mouse contains plant-based foreign body (fb) in bulla exudate (periodic acid-Schiff-stained section). Scale bars: 50 µm in B-F.

The natural flora of the *Jbo*/+ mouse has the potential to influence the outcome of NTHi infection experiments. A requirement for indigenous NP flora in spontaneous OM was investigated by generating germ-free (GF) *Jbo*/+ mice. The incidence of OM in the GF and SPF *Jbo*/*+* mice was the same, but its onset was slightly later, at 5 weeks rather than 4 weeks, in GF conditions. The histological features of OM in GF *Jbo*/*+* mice were inflammatory thickening of the bulla mucosa and accumulation of neutrophil leukocytes and foamy macrophages in the bulla fluid. There were occasional plant-based foreign bodies from bedding or food in the bulla fluid (Fig. 4). This histopathology is indistinguishable from the previously described pattern in SPF *Jbo*/*+* mice (Parkinson et al., 2006; Cheeseman et al., 2011). Thus, normal microbial flora is not an absolute requirement for the *Jbo*/+ mouse to develop OM.

SPF *Junbo* mice were used in our standard NTHi infection experiments. Routine microbiological surveillance of the NP in sentinel mice from our SPF animal facility revealed that the mice were free of all Federation of Laboratory Animal Science Associations (FELASA)-listed mouse pathogens and the presence of the following NP commensals (NP positive culture as a percentage of *n*=1150 mice sampled): *Enterobacter* spp. 2.8%, *Escherichia coli* 2.3%, *Lactobacillus* spp. 0.7%, *Proteus* spp. 22.7%, *Staphylococcus aureus* 62.9%, *Staphylococcus* spp. (not *S.* *aureus*) 56.2%, α-haemolytic *Streptococcus* spp. 94.3% and *Streptococcus* spp. (not β-haemolytic or *Streptococcus* *pneumoniae*) 37.0%. The effect of ME commensal bacteria on NTHi titres in ME bulla fluids was investigated in 12-week-old *Jbo*/*+* mice inoculated with 10^6^ CFU of NTHi 162sr by comparing growth on selective (antibiotic) and non-selective plates. NTHi titres were very similar when ME bulla fluids had pure NTHi cultures (10^4.5^ CFU/µl; 95% CI 10^4.3^-10^4.8^, *n*=59) or mixed cultures with NTHi and *Proteus* or other commensals (10^4.5^ CFU/µl; 95% CI 10^4.3^-10^4.7^, *n*=74).

### Population dynamics of NTHi infection

**Fig. 5.**
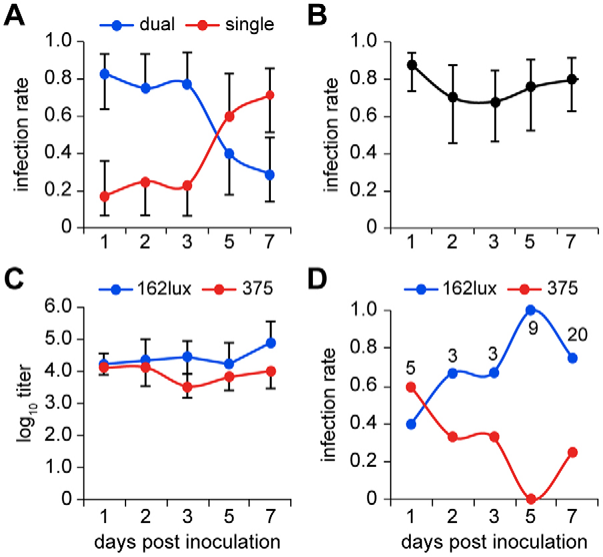
**NTHi monocultures predominate 7 days after co-inoculation with two strains.** *Jbo*/*+* mice were co-inoculated with 5×10^5^ CFU each of NTHi 162lux and NTHi 375. (A) Dual infections predominate on day 1 post infection, but single infections predominate on day 7. (B) The ME infection rate remains high from day 1 to 7. (C) ME titres are comparable for 162lux and 375 in co-infections. Data are represented as mean±95% CI and overlapping error bars are omitted. (D) Single infections with NTHi 162lux predominate at day 7. The number of monocultures is indicated alongside each time point.

When two NTHi strains able to be monitored independently, 162lux and 375, were co-inoculated into 8- to 10-week-old *Jbo*/*+* mice, 83% of ME gave rise to a mixed culture of both strains at day 1, but 71% of ME gave a monoculture of one or the other, but not both, strains at day 7. In this period, ME infection rates remained high, ME titres of each strain were comparable in co-infections, but NTHi 162lux monocultures predominated by day 7 (Fig. 5). Of interest, in at least one mouse the right and left ME were infected by monoculture of the alternative NTHi strains, indicating that each ME can operate as a separate compartment for growth and selection within the same animal.

### *Jbo*/*+* mouse produces an innate immune response in the ME to NTHi infection

**Fig. 6.**
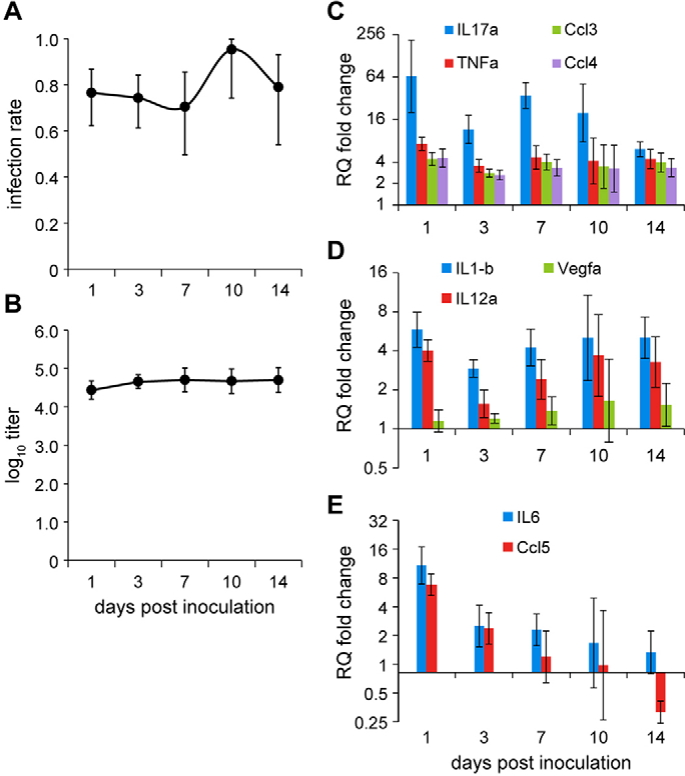
**Host innate immune response in NTHi-infected bulla fluids.** (A,B) ME infection rate (A) and titre (B) in 8-week-old *Jbo*/*+* mice inoculated with 10^6^ CFU of NTHi 375 (*n*=12 per cohort); mean±95% CI. (C-E) Modulation of gene expression in NTHi-infected ME samples relative to GF ME samples detected by RT-qPCR. Each time point represents biological replicate pools of *n*=3-6 NTHi-infected ears and *n*=10 biological replicate pools of GF bulla samples as baseline controls; data are represented as mean relative quantification (RQ)±95% CI.

The host innate immune response was assessed in the ME of 8- to 10-week-old *Jbo*/*+* mice following NTHi infection using 8- to 10-week-old *Jbo*/*+* GF mice as a sterile inflammatory baseline control. We chose to study expression of Toll-like receptor (TLR) response cytokine genes that are likely to be activated by NTHi ligands binding to TLRs (e.g. TLR2, TLR4, TLR9) that are expressed by macrophages, monocytes and neutrophils (http://www.immgen.org) and are relevant to OM and AOM (Juhn et al., 2008; Kaur et al., 2015). We used bulla samples collected from mice at 1, 4, 7 and 14 days post-inoculation with 10^6^ CFU of NTHi 375 when NTHi infection rates were ≥70% (Fig. 6A) and bacterial titres in the range 10^4.4^-10^4.7^ CFU/µl (Fig. 6B). Gene expression patterns varied; *Il17a*, *Tnfa*, *Ccl3* and *Ccl4* showed the highest relative upregulation (four- to 64-fold) throughout this time course relative to GF baseline controls (Fig. 5C); *IL1b* and *IL12a* declined slightly at the day 3 time point but were otherwise moderately elevated (twofold or greater); *Vegf*, a marker for hypoxia in the inflammatory environment, did not change (twofold or less; Fig. 6D); and *Ccl5* and *Il6* were elevated at day 1 but declined to baseline levels by day 14 (Fig. 6E).

The combined data from NTHi infections indicate that the *Jbo*/*+* mouse is an important new infection model that can facilitate study of NTHi pathogenesis and OM disease. ME infection of mice is achieved efficiently by a single IN inoculation with NTHi, producing high rates of ME infection and bacterial titres in bulla fluids that are sustained in a proportion of ME up to 56 days post-inoculation. NTHi coexists with indigenous bacteria in SPF mice but still induces a host innate response following infection. Having established a robust NTHi infection model for the study of OM pathogenesis, we then explored its potential translational utility in challenge (vaccine) studies and treatment (antimicrobial) studies.

### An adaptive immune response in the *Jbo*/*+* mouse is protective against NTHi challenge with a homologous NTHi strain

**Fig. 7.**
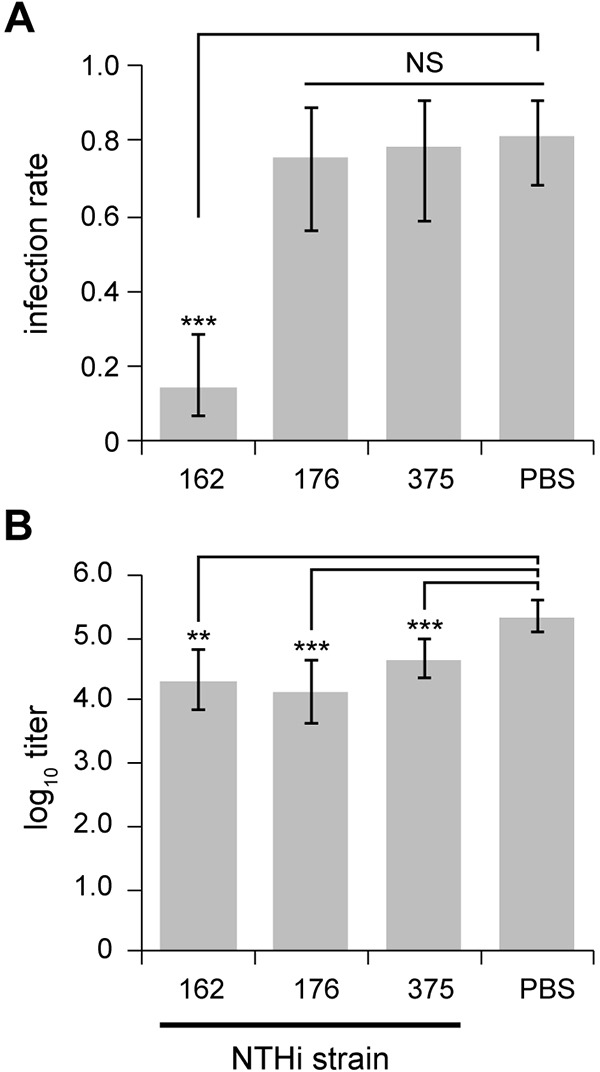
**Homologous and heterologous protection for mice immunized with killed NTHi bacteria.** (A) *Jbo*/*+* mice immunized three times subcutaneously with 10^8^ killed NTHi 162 (but not 176 or 375) had a significantly lower ME infection rate when inoculated with 10^6^ CFU of NTHi 162sr compared with PBS-immunized controls. (B) *Jbo*/*+* mice immunized with killed NTHi 162, 176 or 375 had significantly lower ME titres than PBS-immunized controls. *n*=14-15 mice per cohort for NTHi 176- and 375-immunized mice and *n*=30 for NTHi 162- and PBS-immunized mice; data are represented as mean±95% CI. (A) Fisher’s exact tests comparing infection rates for each NTHi strain with the PBS control. (B) *t*-tests comparing titres for each NTHi strain with the PBS control. NS, not significant; **P<0.01; ***P<0.001.

Following the advent of multivalent pneumococcal vaccines, NTHi is now the leading cause of OM (Benninger, 2008), but currently there are no effective vaccines against this bacterium. An initial assessment of the *Jbo*/*+* mouse as a model for vaccine studies was made by immunizing 5-week-old *Jbo*/*+* mice subcutaneously with killed NTHi 162. At 12 weeks, mice were inoculated IN with live NTHi 162sr, the homologous strain. Terminal assessment at day 7 post-inoculation revealed significantly reduced ME infection rates compared with PBS-immunized control animals (15 versus 81%, respectively, *P*=9.15×10^−12^; Fig. 7A). In mice that were NTHi culture positive, ME infection was unilateral and titres were significantly lower than those in PBS-immunized controls (*P*=1.41×10^−3^; Fig. 7B). For mice that were immunized with killed heterologous NTHi strains 176 or 375, then inoculated IN with live strain 162sr, there was no significant protection indicated by ME infection rates; however, bacterial titres were ∼1 log_10_ lower compared with PBS-immunized controls (*P*=0.00015 and *P*=0.0011 for 176 and 375, respectively; Fig. 7A,B). Thus, the antibody response against NTHi in the *Jbo*/+ mouse can provide protection and can discriminate between homologous and heterologous NTHi challenge.

### Azithromycin treatment eliminates NTHi ME infection

**Fig. 8.**
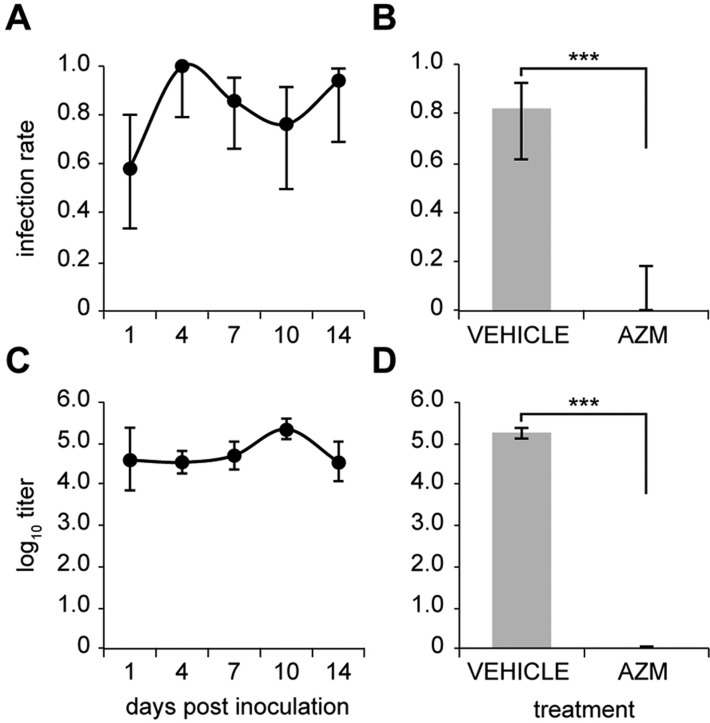
**Azithromycin treatment eliminates NTHi infection.** (A,C) Time course of ME infection rate (A) and titre (C) for 8-week-old *Jbo*/*+* mice inoculated with 10^6^ CFU of NTHi 162 (*n*=11-12 per time point). (B,D) *Jbo*/*+* mice were treated with 100 mg/kg azithromycin (AZM) by oral gavage once a day for 3 days starting on day 4 post-inoculation with 10^6^ CFU of NTHi 162, and sampled at day 7. (B) ME infection rate was significantly reduced in the AZM-treated group. (D) NTHi titre in the vehicle-treated group was 10^5.3^ CFU/µl (10^5.1^-10^5.4^), whereas NTHi were not detected in the AZM-treated group (detection limit 10^2^ CFU/µl); *n*=15 *Jbo*/*+* per group. Data are represented as mean±95% CI. (B) Fisher’s exact test. (D) One-sample *t*-test using a hypothetical sample mean of 10^2^ CFU/µl. ***P<0.001.

Antibiotic resistance in the clinical setting is on the increase for commensal pathogens (Benninger, 2008); this has the potential for impact on treatment regimens for disease caused by NTHi, including OM. To make a preliminary assessment of the *Jbo*/+ mouse model for testing new antimicrobials or antibiotic delivery strategies, we treated 8-week-old *Jbo*/+ mice with a 3 day course of azithromycin (100 mg/kg) starting at day 4 post-inoculation with NTHi 162. Compared with untreated mice, in which ME infection was detected on day 1 post-inoculation and was maintained in ≥80% of ME at titres of 10^4^-10^5^ CFU/µl from day 4 to 14 (Fig. 8A,C), NTHi had been eliminated from all ME bulla fluids of azithromycin-treated mice (Fig. 8B,D). These data provide initial evidence of the potential for the *Jbo*/+ mouse to be used for efficient screening of antimicrobial agents or treatment regimens for OM.

### IN challenge with the pneumococcus

To investigate whether ME infection could be achieved with the other major human otopathogen, 8-week-old *Jbo*/*+* mice were inoculated IN with 10^5^ CFU of *S. pneumoniae* strain D39 in 10 µl (supplied by Peter Andrew, University of Leicester, UK; Haste et al., 2014). One day post-inoculation, the ME infection rate (63%) and titres (10^4.2^ CFU/µl, 95% CI 10^3.4^–10^4.9^, *n*=15) were similar to those seen following NTHi challenge. On day 2, however, unlike with NTHi, there were unexpected deaths in five of 15 mice with this relatively low dose and a small volume that would not be expected to infect the lower airway. The experiment was immediately terminated, and retro-orbital blood samples from three of 10 survivors indicated bacteraemia. In the surviving mice, the ME infection rate was 50% and titres of 10^4.5^ CFU/µl (95% CI 10^2.8^–10^6.2^, *n*=10) were attained. Thus, the *Jbo*/*+* infection model can support ME pathogens other than NTHi, but for the pneumococcus this needs to be explored further using appropriately attenuated strains with reduced virulence.

## DISCUSSION

A recent review of the use of animals in OM research concluded that there is a need to improve our current animal models, develop new ones and use a diversity of species to ensure that differences between any one species and humans do not bias our data (Li et al., 2013). In this work, we show that *Junbo*, a mutant mouse line that develops OM spontaneously, has significant potential for ME infection studies because it can be reproducibly infected with a single IN inoculum of NTHi. NTHi rapidly ascends the ET and colonizes the ME. NTHi infection is detected in bulla fluids at 1 h, titres peaked between day 7 and 14, and 20% of individual ME were infected up to day 56. Mouse ME bacterial titres (10^4^-10^5^ CFU/µl) are comparable to those reported in direct inoculation of the chinchilla ME (10^6^-10^7^ CFU/ml) albeit that the volume of material recovered from the mouse is substantially less. Infection in the chinchilla was cleared by day 35 using the same NTHi strain set used in our study (Bouchet et al., 2003).

The *Junbo* infection model provides a number of new perspectives on OM pathogenesis. In keeping with other animal models, the ME of wild-type mice is not efficiently infected by NTHi following IN inoculation. Microspheres that are similar in size to NTHi are readily translocated into the ET in both wild-type and *Junbo* mice and by this measure there is no obvious deficit in ET barrier function in *Junbo* mice. Other studies have shown that co-infection with viruses, such as adenovirus, promotes NTHi infection in the chinchilla ME (Bakaletz, 2009), and influenza virus promotes pneumococcus ascension of the ET (McCullers et al., 2007) and ME infection (Short et al., 2013). Taken together, these findings suggest that coincident inflammation is a predisposing factor that promotes successful ME bacterial colonization and infection via direct epithelial injury and/or increased mucosal barrier permeability, leading to ME fluid accumulation and creation of a new niche for colonizing bacteria. The hypoxic inflamed ME in *Junbo* mice (Cheeseman et al., 2011) might favour the growth of microaerophilic bacteria, such as NTHi.

The strong association between the incidence of ME and NP infection, the high number of NTHi in the ME relative to NP and exudate along the length of the ET suggests that the ME can act as a reservoir for NP reinfection. The implication is that the ME compartment is not an evolutionary dead end for NTHi. The ME niche will impose strong selection pressure through altered nutrient, oxygen and host defence parameters compared with the NP environment, factors consistent with the evolution of a repertoire of adaptive gene expression, for example the phase variation characteristic of NTHi strains.

The generation of GF *Junbo* mice revealed that bacteria are not necessary for OM to develop but accelerate its onset. The presence of foreign bodies in histological sections of bulla fluids shows that fine particulate matter can gain access to the ME via the ET. This suggests that physical irritants, which might include exposure to ammonia in laboratory mice, can act as inflammatory stimuli. The importance of non-microbial inflammatory stimuli in OM is not surprising given the established role of cigarette smoke as a risk factor for OM (Cayé-Thomasen et al., 2013).

Perturbation of innate immunity in the *Junbo* mouse via impaired Evi1 negative regulation of nuclear factor-κB (Xu et al., 2012) is likely to contribute to the inflammatory OM phenotype and susceptibility to NTHi infection. In this regard, *Junbo* has similarities to other mouse mutants with innate immunity deficits (Leichtle et al., 2011) where inflammation and bacterial clearance following intrabulla challenge are prolonged in comparison to immune-competent inbred strains, such as C57BL/6 (Ryan et al., 2006).

The global activation of an innate immune response following introduction of NTHi directly into the naïve mouse ME bulla has been characterized in a recent infection study. Bulla inflammatory cells and mucosal hyperplasia and inflammation peaked at 2 days and returned to baseline by 7 days. Mucosal gene expression showed a temporal pattern of gene clusters associated with neutrophil and macrophage recruitment and activation, leading to resolution. It was suggested that dysregulation of pro- and anti-inflammatory signalling might be an important feature of chronic or recurrent OM (Hernandez et al., 2015).

NTHi express a number of ligands that might be recognized by different TLRs. In the context of experimental OM in the mouse, membrane-associated NTHi ligands, such as peptidoglycan and peptidoglycan-associated proteins (e.g. P6), are TLR2 ligands, and lipopolysaccharide activates TLR2 and TLR4 signalling (Leichtle et al., 2009). Destruction of NTHi in the OM lesion is likely to release bacterial DNA and CpG motifs that are ligands for TLR9 (Leichtle et al., 2012), and bacterial RNAs (see below) are ligands for TLR7 and TLR8. In children with AOM, TLR2 and TLR4 are significantly upregulated in bacterial culture-positive bulla fluids compared with culture-negative bulla fluids (Kaur et al., 2015). These TLRs signal through MyD88 to upregulate pro-inflammatory cytokines.

In the *Junbo* mouse, antigen-presenting cells capable of recognizing bacterial ligands for TLRs are present in the bulla fluids at the initiation of infection, and the bulla fluids were culture positive for NTHi throughout the 14 day time course of the experiment. We cannot exclude the possibility that TLR ligands from endemic bacteria contribute to expression of response cytokines, but we were able to exclude this variable from the baseline control by using bulla fluids from sterile inflammation in GF *Junbo* mice. In NTHi-challenged *Junbo* mice, there is upregulation of *Tnfa*, *Il1b*, *Il12a*, *Ccl5* and *Il6*, which are implicated as inflammatory mediators in OM (Juhn et al., 2008). Furthermore, *Tnfa*, *Il1b*, *Il6*, *Ccl3* and *Ccl4* are differentially expressed in culture-positive compared with culture-negative middle ear fluids from children with AOM (Kaur et al., 2015). IL17a is important in neutrophil inflammation in the exacerbation by NTHi of chronic obstructive pulmonary disease (Roos et al., 2015). Expression of *Il6* and *Ccl5* declined after initial NTHi infection, whereas other cytokines showed sustained upregulation. *Vegfa* is a marker of chronic inflammatory hypoxia in *Junbo* OM (Cheeseman et al., 2011), but *Vegfa* expression was unaltered by NTHi infection. These results indicate that NTHi infection stimulates a host innate immune response and further elevates cytokine gene expression, but that NTHi infection itself does not exacerbate hypoxia and hypoxia-inducible factor signalling.

By 7 days post-inoculation, NTHi infection is well established in the *Junbo* mouse ME, and histology reveals an abscess-like structure with a core of necrotic neutrophils surrounded by viable and apoptotic neutrophils and foamy macrophages. The exudate neutrophil density is ∼7×10^6^/µl (Cheeseman et al., 2011) and 1-2 logs higher than viable NTHi (10^4^-10^5^ CFU/µl). We interpret the punctate and aggregate NTHi hybridization signals as intact single or clustered bacteria, or RNA released from lysed bacteria. Pus consists of neutrophils surrounded by neutrophil extracellular nets formed when the cell and nuclear membranes break down and release chromatin (Brinkmann and Zychlinsky, 2012). The extracellular histones formed in this process can have powerful antimicrobial effects and stimulate sterile inflammation via activation of the NLRP3 inflammasome (Allam et al., 2014). The impact of the host innate immune response on NTHi population dynamics will be a key determinant of changes in host-microbe interaction and disease and requires further investigation. Also, there are major implications concerning the extreme bottleneck occurring in the pathogenesis of OM, indicated by the monoclonal cultures (strain 375 or 162lux but not both) of ME exudate. Once initiated, ME infection rates remain high up to 14 days post-inoculation; it is not known whether NTHi titres were held in check by the immune response and/or whether other mechanisms, such as host sequestration of nutrients, for example iron (Szelestey et al., 2013), contribute to limiting NTHi population growth. Unlike the infected ME of the chinchilla, where NTHi is known to form a biofilm, our immunohistochemical and *in situ* hybridization analyses provided no clear evidence consistent with this mode of bacterial growth in the *Junbo* mouse ME bulla. It is likely that the inflamed hypoxic environment and the high incidence of other microbial flora that can exist in the *Junbo* mouse ME present a substantially different growth challenge for NTHi from that provided when infecting the sterile chinchilla ME (Jurcisek and Bakaletz, 2007; Brockson et al., 2014). The maintenance of NTHi infection in the mouse for periods of at least 8 weeks provides an opportunity to carry out longitudinal infection studies whereby the host response and concomitant microbial within-host adaptation and population dynamics can be studied and compared over time within and between individual mice.

Having established the basic dynamics of NTHi infection in the *Junbo* mouse, we carried out two translational studies using quantitative end points. We have shown significant protection of the *Junbo* mouse following immunization with killed NTHi bacteria and subsequent IN challenge with the homologous NTHi strain. *Junbo* mice immunized with heterologous NTHi strains and subsequently infected with NTHi 162sr failed to show protection as indicated by infection rate, but some reduction in ME titre was observed. This is likely to reflect the substantial genetic and phenotypic heterogeneity that exists within natural populations of NTHi and the implicit challenge in developing a vaccine against NTHi OM and other diseases. These data offer proof in principle that the *Junbo* infection model could be used for relatively high-throughput screening of candidate NTHi vaccine antigens. In clinical practice, antibiotic resistance is on the increase for NTHi and will potentially have a major impact on OM treatment regimens. The *Junbo* mouse model has demonstrated potential utility for investigating improved antibiotic treatments. A 3 day treatment of NTHi-infected mice with systemic azithromycin cleared ME infection, opening the possibility for the model to be used either to screen novel antibiotics for treatment or to design improved regimens and routes of delivery. An important feature of the *Junbo* OM model for future studies is the inflammatory thickening of the TM (Parkinson et al., 2006), as seen in affected humans (Berger et al., 1996), making it a suitable model for testing trans-TM delivery of antibiotics.

In summary, mouse models offer the general advantages of relatively low costs, better availability of immunological reagents, and ease of control of host genetics and microbial status. In the present study, we tested three mouse lines, *Junbo*, *Tgif* and *Jeff*, each with a characterized mutation mediating a different susceptibility to NTHi infection. Several genes associated with increased susceptibility to spontaneous OM in the mouse have now been shown to be relevant to human disease through candidate gene studies in family-based cohorts (Rye et al., 2010). As new candidate human OM disease genes are discovered, their role in NTHi susceptibility could be investigated through the use of genetically modified mouse models. The high efficiency and reproducibility of IN inoculation in the *Junbo* mouse provides a robust platform to investigate aspects of host-microbial interaction and the innate immune response to NTHi, and to test an adaptive immune response and antibiotic treatment against NTHi, which have wide translational potential for clinical intervention. The *Junbo* model uses a non-invasive IN challenge route and a single terminal assessment, rather than resampling the same animal, and therefore represents a welfare refinement.

## MATERIALS AND METHODS

### Ethics statement

Full details of these studies were reviewed and approved by the Medical Research Council (MRC) Harwell ethical review committee. The humane care and use of mice in this study was carried out under the authority of the appropriate UK Home Office Project Licence.

### Mouse strains

The majority of experiments used *Junbo* mice that were congenic on a C3H/HeH genetic background (Parkinson et al., 2006), and this mouse strain is available from the European Mouse Mutant Archive (EM:00091). For non-academic groups, the *Junbo* model is available through MRC Technology (http://www.licensingopportunities.co.uk/research-tools/79/otitis-media/).

For comparative purposes, *Junbo* mice congenic on a BALB/c background were used in other infection studies. *Tgif* mice were congenic on a C57BL/6J background (Tateossian et al., 2013), and *Jeff* mice (EM:00375; Hardisty-Hughes et al., 2006) were on a mixed C3H/HeH and C57BL/6J genetic background.

### Husbandry and microbiological surveillance of SPF *Junbo*, *Tgif* and *Jeff* mice

SPF mice were housed in individually ventilated cages (Tecniplast) under a 12 h light-12 h dark cycle, temperature of 21±2°C and humidity of 55±10%, on autoclaved Datesand grade 6 pine bedding. Mice were fed an irradiated expanded RM3 diet (Special Diets Services, Witham, UK) and given water *ad libitum*. Air changes were 70-75 per hour. Microbiological surveillance of sentinel mice from our SPF animal facility was performed by Harlan laboratories following the FELASA screening guidelines.

SPF mice that were challenged with bacteria were housed in a Biological Containment Unit IVC rack (Allentown) operated at negative pressure with an exhaust rate of 35.7 m^3^ per hour.

### Generation and microbiological surveillance of GF *Junbo* mice

Germ-free C3H/HeJ stock was obtained from the CDTA-CNRS (Orleans, France). GF *Junbo* and C3H/HeH mice were re-derived by hysterectomy on embryonic day 19.5 of pregnancy to establish a breeding colony. GF mice were reared on irradiated Alpha-Dri substrate (SPP) plus shredded paper bedding and were housed in wire-topped cages within sterile flexible film isolators (Harlan-Isotec) fitted with DPTE alpha ports (La Calhene). GF mice were provided with irradiated RM3 diet and autoclaved water *ad libitum*.

The isolators were maintained under positive pressure (5-10 mm water) operated at 12-15 air changes per hour. Faeces, urine and bedding were tested every 4 weeks for the presence of fungi, anaerobic and aerobic bacteria using Sabouraud media (Becton Dickinson), thioglycolate media (Becton Dickinson) and nutrient broth (Becton Dickinson), respectively.

### Histology, immunohistochemistry and *in situ* hybridization

Middle ear histology in GF and SPF mice was assessed in Haematoxylin- and Eosin-stained 3-µm-thick wax sections as previously described (Cheeseman et al., 2011).

To examine the histology of NTHi-infected bullae by immunohistochemistry (*n*=6) or by *in situ* hybridization and lesion profiling, 11-week-old *Jbo*/*+* mice (*n*=6) were inoculated IN with 10^6^ CFU NTHi 162, and heads collected 7 days post-inoculation were fixed for 48 h in neutral buffered formalin. The heads of non-challenged *Jbo*/*+* mice (*n*=5) collected at the same time served as negative controls. For immunohistochemistry, EDTA decalcification was 5-7 days. To maximize RNA integrity for *in situ* work, a band saw was used to isolate the bullae from fixed heads and EDTA decalcification achieved in 48 h.

For immunohistochemistry, 4-µm-thick wax sections were cut onto electrostatically charged slides and dried overnight at 37°C before a final drying at 60°C for 25 min. Sections were de-waxed in xylene, hydrated through ethanol and washed three times in Tris buffer. Endogenous peroxidase was blocked using Dako REAL peroxidase blocker (S2023) for 10 min following antibody incubations.

Rat monoclonal anti-F4/80 (Serotec; MCA497G) was used to detect macrophages. Antigen retrieval was performed using Dako proteinase K (S3020) for 20 min at room temperature. The antibody was diluted 1:400 and incubated for 30 min at room temperature. Secondary detection was carried out using the Vector Labs ImmPress HRP anti rat kit (MP-744-15) according to the manufacturer's instructions. Rabbit polyclonal anti-cleaved caspase 3 (Abcam; ab2302) was used to detect apoptotic cells, and rabbit polyclonal anti-histone 3 (Abcam; ab61251) to detect histones. Antigen retrieval was carried out using Vector high-pH antigen retrieval reagent at 60°C for 12 h. The rabbit polyclonal antibodies were diluted 1:20 or 1:50 for caspase 3 and histone 3, respectively, and incubated for 60 min at room temperature. Secondary detection was carried out using Dako (K4011) Envision+ System HRP anti-rabbit according to the manufacturer's instructions. Antibodies were diluted in Dako antibody diluent (S0809), and negative controls were carried out using the antibody diluent alone. Visualization was achieved using Dako (K3468) liquid DAB+ substrate chromogen system. Counterstaining was carried out using Harris Haematoxylin prior to dehydration through ethanol, clearing in xylene and mounting in Clearview mountant (Thermo Fisher Scientific).

For lesion profiling, bright-field images of Haematoxylin- and Eosin-stained sections were acquired using a Hamamatsu NanoZoomer slide scanner, and the morphometric measurements were made using NanoZoomer software. The average thickness of the mucosa lining the medial surface of the bulla (avoiding the cochlea and the region close to the Eustachian tube) was calculated by dividing the area of mucosa overlying a delineated ∼1000 µm length of supporting bulla bone. The proportion of bulla space occupied by exudate was calculated by dividing the exudate area by the area bounded by the bulla mucosal surface and the tympanic membrane. The treatment groups were blinded for the slide analysis.

*In situ* hybridization was performed on 4-µm-thick wax sections of representative NTHi-challenged (*n*=2) and non-challenged (*n*=2) *Jbo*/*+* mice heads using probe B-HInfluenzae-NTHi375-16SrRNA according to the manufacturer's instructions (Advanced Cell Diagnostic) using the HRP visualization kit. A positive control for RNA integrity (PpiB) and a negative hybridization control (DapB; Advanced Cell Diagnostic) were used.

### Intranasal administration of FITC microspheres

Nine-week-old *Jbo*/*+* (*n*=6) and wild-type littermate (*n*=9) mice were anaesthetized with isofluorane and ∼10^8^ FITC-microsphere beads [1.0 µm blue-green fluorescent (430/465) polystyrene microspheres; Life Technologies] administered IN by applying 5 µl of suspension to each nostril. The mice were euthanized at ∼5 min using a rising concentration of CO_2_ and the heads fixed in neutral buffered formalin. EDTA-decalcified heads were prepared for cryostat sectioning of the ET. Ten-micrometre-thick coronal serial sections of the ET were mounted on consecutive slides. A representative slide with range of ET levels was counterstained with DAPI and mounted in Prolong antifade reagent (Molecular Probes). The sections were scanned, and the number of FITC microspheres were counted in representative levels of each ET and expressed per millimetre of ET profile. The mouse genotypes were blinded for the slide analysis.

### Bacterial strains for inoculum preparation and immunization

NTHi strains used in the study are well characterized, phylogenetically distinct strains (162, 176, 285, 375, 486, 1124 and 1158) from human OM (Cody et al., 2003), and 2019. Growth of indigenous commensal bacterial flora, particularly of *Proteus* spp., rendered a proportion of bacterial count plates unreadable. For this reason, NTHi strains expressing resistance to streptomycin or kanamycin (e.g. NTHi 162sr or 162kr) were generated. Spontaneous streptomycin-resistant colonies were selected after plating bacteria at high density (2×10^10^ CFU per plate) on media containing streptomycin (300 µg/ml). Strain 162kr was made by insertion of a kanamycin resistance cassette onto a position in the NTHi genome (HI0227) that had no characterized phenotype. These strains enabled antibiotic selection of NTHi during culture and counter-selection against any indigenous bacteria; this increased the number of ME providing quantitative data during experiments.

Strain NTHi 162lux was constructed by transforming strain 162 with the *luxCDABE* genes using chromosomal DNA isolated from *H. influenzae* strain Xen21 (Caliper Life Sciences).

Bacteria from −80°C stocks were grown overnight at 37°C in air supplemented with 5% CO_2_ on Brain Heart Infusion-Levinthals (BHI-Lev) agar, then used to inoculate BHI-Lev broth and grown to log-phase before preparing the animal inoculum. The inoculum size was calculated from optical density (OD) measurement using the conversion factor that an OD *A*_490_ of 0.4=1×10^9^ CFU/ml. Bacteria were pelleted by centrifugation at 13,000 ***g*** for 3 min, then resuspended to 10^10^ CFU/ml in PBS containing 2% gelatin. The titre of each inoculum was assessed pre- and post-inoculation by dilution and plating.

For subcutaneous immunization of mice, bacteria from liquid culture were pelleted, resuspended in PBS, then killed by treatment with 1% paraformaldehyde (1 h at 37°C, then overnight at 4°C); loss of viability was confirmed by plating. Killed NTHi were stored in 1% paraformaldehyde for up to 5 weeks at 4°C and their integrity was assessed by phase contrast microscopy. Before use, the bacteria were pelleted by centrifugation at 13,000 ***g*** for 3 min and resuspended to give 10^10^ CFU/ml in PBS, then mixed with Adjuplex (Sigma) adjuvant according to the manufacturer's instructions.

### Intranasal challenge

SPF mice aged either 8±1 or 12±1 weeks were anaesthetized with isofluorane, and 5 µl of the 10 µl inoculum was applied to each nostril. For each time point in an experiment, mice in cohorts of 11-15 animals were inoculated with doses ranging from 10^3^ to 10^8^ CFU.

### Terminal sampling of blood, bulla fluids and nasopharynx

Bulla fluids were sampled terminally via the TM as previously described (Cheeseman et al., 2011), and the volume was estimated using a 0-2 µl filtered pipette tip (average volume 0.5 µl, range 0.1-1.25 µl), then transferred into 500 µl of PBS. In wild-type mice and in a proportion of *Jbo*/*+* ME, the TM was clear and there was no detectable bulla fluid. These bullae were washed twice with 2 µl of sterile PBS and the washings added back to 500 µl of PBS.

In some experiments, the NP was sampled after collecting bulla fluids by washing with 200 µl PBS introduced into the NP opening on the palate and collecting the wash fluid from the nares. To ensure that this order of collection did not displace ME NTHi and bias NP numbers recovered, the NP was sampled first in one cohort of *n*=15 mice; the infection rate was found to be not significantly different (67 versus 80%, *P*=0.6816, Fisher’s exact test, *n*=15 mice per group) and the titres comparable when the NP was sampled either before or after the ME (10^1.1^ and 10^1.6^, respectively).

### NTHi culture of ME bulla fluids and NP wash

Bulla fluids and NP washes in PBS were mixed by three 10 s bursts on a vortex mixer, after which 10-fold dilutions (10^−1^, 10^−2^) were made in PBS. Fifty microlitres of each ME dilution or NP wash was spread on a BHI-Lev agar plate. The detection limit was 10 CFU/µl for the primary bulla fluid preparation and 100 CFU/µl for the 10^−1^ dilution. The primary bulla fluid suspension was centrifuged at 13,000 ***g*** for 3 min and the pellet frozen on dry ice, then stored at −80°C for RNA isolation (see below).

In experiments using antibiotic-resistant strains, bulla samples were plated on media supplemented with 300 µg/ml streptomycin or 30 µg/ml kanamycin. The ME commensal bacteria were assessed using a non-selective plate in parallel. Plates were cultured overnight at 37°C to calculate NTHi titres. Representative NTHi colonies were examined by phase contrast microscopy to confirm its small coccobacillus morphology.

In the co-infection experiment with NTHi 162lux and 375 (5×10^5^ CFU of each strain IN), a monoculture is defined as ≥10 colonies, all of the same strain (either 162lux or 375), on the BHI primary culture plate.

### Infection rates and bulla fluid titre

To compare ME infection rates, we used the following index of infected bulla fluids:
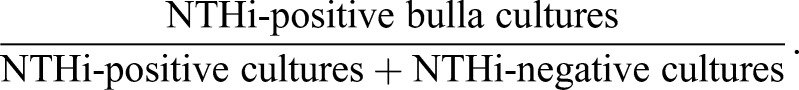


The following two categories were excluded from this index: PBS bulla washes (because they yielded a very low percentage with NTHi growth); and, in experiments using non-antibiotic-resistant NTHi strains, bulla fluids that gave *Proteus* overgrowth preventing NTHi detection. The ME NTHi titre (in colony-forming units per microlitre) was calculated from plate counts, sample dilution and bulla fluid volume.

### Bioluminescent imaging

Mice were inoculated with NTHi 162lux, and bioluminescent signals from the head (oral cavity aspect of the palate after dissecting away the mandible) or, for dual infection studies, the proportion of NTHi colonies on the BHI ME culture plates were imaged using an IVIS Lumina II system (Perkin Elmer).

### Real-time quantitative PCR (RT-qPCR) of bulla fluids

Eight-week-old SPF *Jbo*/*+* mice were inoculated with 10^6^ CFU NTHi 375, and cohorts were sampled terminally at days 1, 3, 7, 10 and 14 post-inoculation. Cell pellets (*n*=4-6) were pooled from bulla fluids that yielded NTHi monocultures on the 10^−1^ dilution plate (equivalent to <100 CFU/µl commensal bacterial), and for each time point there were *n*=3-6 biological replicate pools. Bulla fluids from 8- to 10-week-old GF *Jbo*/*+* mice were collected into 20 µl of RNase-free water for RNA isolation and used as a baseline expression control. Each GF pool comprised *n*=4-6 bulla fluids, and there were *n*=10 biological replicates. RNA extraction, cDNA synthesis and RT-qPCR TaqMan were performed as previously described (Cheeseman et al., 2011) for *Ccl3*, *Ccl4*, *Ccl5*, *Il1b*, *Il6*, *Il12a*, *Il17a*, *Tnfa* and *Vegfa*. RT-qPCR was performed in triplicate technical assays. Data were normalized using *Hrpt1* and β-actin as endogenous controls. Fold changes of expression (ddCts) of NTHi-infected bulla fluid white blood cells over non-infected white blood cells were calculated using AB 7500 software v2.0.1 and expressed as mean relative quantification (RQ)±min/max, with error bars representing 95% CI.

### Immunization with NTHi bacteria

Each *Jbo*/*+* mouse received three subcutaneous immunizations in the intrascapular skin with 10^8^ CFU of killed bacteria and adjuvant in 50 µl. Immunizations were at the age of 5, 8 and 10 weeks. Mice were immunized with NTHi 162sr, 176 or 375, after which they were inoculated IN at 12 weeks with 10^6^ CFU NTHi 162sr. ME infection was assessed at 7 days post-inoculation.

### Azithromycin treatment of NTHi infection

Eight-week-old *Jbo*/*+* mice were treated using a 3 day course of 100 mg/kg azithromycin delivered once a day by oral gavage. Treatment started at day 4 after IN inoculation with 10^6^ CFU NTHi 162, and bulla fluids were sampled on day 7. A sham-treated control group was gavaged with vehicle alone (2% methoxycellulose solution). The minimal inhibitory concentration for NTHi 162 was determined to be 1 µg/ml.

### Statistical analysis

Log_10_-normalized NTHi titres were analysed using *t*-tests (mouse strain susceptibility data, immunization data and antimicrobial data) or by one-way ANOVAs and Tukey’s multiple comparison tests for *post hoc* testing (inoculation dose data and 24 h time course data). ME mucosal thickness, bulla fluid area proportions and microsphere counts per millimetre of ET were analysed with a Mann–Whitney *U*-test. Fisher’s exact and χ^2^ tests were used to analyse frequency data (TM phenotypes, occurrence of bulla fluids, ME and NP infection rates). Data are presented as the mean±s.e.m., mean±95% CI or median±95% CI. Two-tailed test values *P*<0.05 were considered significant.
